# Mood shapes the impact of reward on perceived fatigue from listening

**DOI:** 10.1177/17470218241242260

**Published:** 2024-04-23

**Authors:** Ronan McGarrigle, Sarah Knight, Lyndon Rakusen, Sven Mattys

**Affiliations:** 1 Department of Psychology, University of Bradford, Bradford, UK; 2 Department of Psychology, University of York, York, UK

**Keywords:** Listening-related fatigue, effortful listening, motivation, reward, auditory attention, dichotic listening, speech perception

## Abstract

Knowledge of the underlying mechanisms of effortful listening could help to reduce cases of social withdrawal and mitigate fatigue, especially in older adults. However, the relationship between transient effort and longer term fatigue is likely to be more complex than originally thought. Here, we manipulated the presence/absence of monetary reward to examine the role of motivation and mood state in governing changes in perceived effort and fatigue from listening. In an online study, 185 participants were randomly assigned to either a “reward” (*n* = 91) or “no-reward” (*n* = 94) group and completed a dichotic listening task along with a series of questionnaires assessing changes over time in perceived effort, mood, and fatigue. Effort ratings were higher overall in the reward group, yet fatigue ratings in that group showed a shallower linear increase over time. Mediation analysis revealed an indirect effect of reward on fatigue ratings via perceived mood state; reward induced a more positive mood state which was associated with reduced fatigue. These results suggest that: (1) listening conditions rated as more “effortful” may be *less* fatiguing if the effort is deemed worthwhile, and (2) alterations to one’s mood state represent a potential mechanism by which fatigue may be elicited during unrewarding listening situations.

## Introduction

Fatigue from mental exertion is a familiar subjective experience for most individuals. In most cases, this experience is transient and does not have lasting negative consequences. However, for some individuals (e.g., those with chronic conditions like cancer and diabetes), the effects of mental fatigue may be more pronounced and potentially debilitating (Bryant et al., 2004; Hockey, 2013). As well as compromising wellbeing, mental fatigue has been shown to disrupt an individual’s ability to perform a wide range of tasks (Herlambang et al., 2021; Marcora et al., 2009), and may result in safety issues like increased likelihood of traffic accidents (Ting et al., 2008). Theoretical approaches highlight the roles of cognitive resource depletion (Craig & Klein, 2019; Gergelyfi et al., 2015) and motivation (Herlambang et al., 2019) in determining the experience of mental fatigue. Hockey’s (2013) Motivational Control Theory (MCT) proposes that fatigue is an adaptive emotional response to conflict that arises in everyday life due to competing demands and priorities. In other words, we experience fatigue as an evolutionarily adaptive response to signal that a particular task or goal is no longer worth the investment of cognitive effort.

Interest in the mental fatigue that arises from effortful speech understanding has increased rapidly in recent years, with recent evidence revealing associations between hearing loss and fatigue (Alhanbali et al., 2017; Davis et al., 2021; Holman et al., 2019; Hornsby & Kipp, 2016). Understanding speech, even for normal-hearing listeners, can tax cognitive resources due to the presence of background noise and other forms of distraction during everyday communication (Mattys et al., 2012). Although the link between repeated episodes of effortful listening and longer term fatigue makes intuitive sense (McGarrigle et al., 2014), the relationship between perceived effort and fatigue appears more complex than originally conceived (Herrmann & Johnsrude, 2020; McGarrigle & Mattys, 2023; Pichora-Fuller et al., 2016). In particular, fatigue may accumulate independently of perceived effort (McGarrigle, Rakusen, & Mattys, 2021), or vice versa (Alhanbali et al., 2023). Although perceived effort is often seen as a proxy for performance estimation (Moore & Picou, 2018), fatigue is determined at least partly by one’s affective state (van der Linden et al., 2003). Indeed, in the context of speech perception, heightened daily life experiences of listening-related fatigue have been shown to be associated with an individual’s level of mood disturbance (McGarrigle, Knight, et al., 2021).

The Framework for Understanding Effortful Listening (FUEL) proposes that listening-related effort and fatigue may be influenced by one’s state of motivational arousal (Pichora-Fuller et al., 2016). Studies to date have generally focused on the effects of reward-based motivation on perceived (i.e., self-reported), behavioural, and/or physiological measures of effort allocated (Carolan et al., 2021; Koelewijn et al., 2018; Richter, 2016). These studies have revealed mixed findings. Koelewijn et al. (2018) examined the effect of monetary reward (high/low) on the task-evoked pupil response (a physiological marker of cognitive effort) and self-reported indices of effortful listening in normal-hearing young adults. As predicted, the task-evoked pupil response was larger (indicating increased resource allocation) in the high than low reward condition. However, there was no effect of reward on perceived effort. Carolan et al. (2021) also manipulated reward amount in a sample of young normal-hearing adults. In their study, however, effort ratings were higher when the monetary reward was higher, suggesting that the additional monetary incentive translated into an increase in perceived effort.

**Figure 1.**
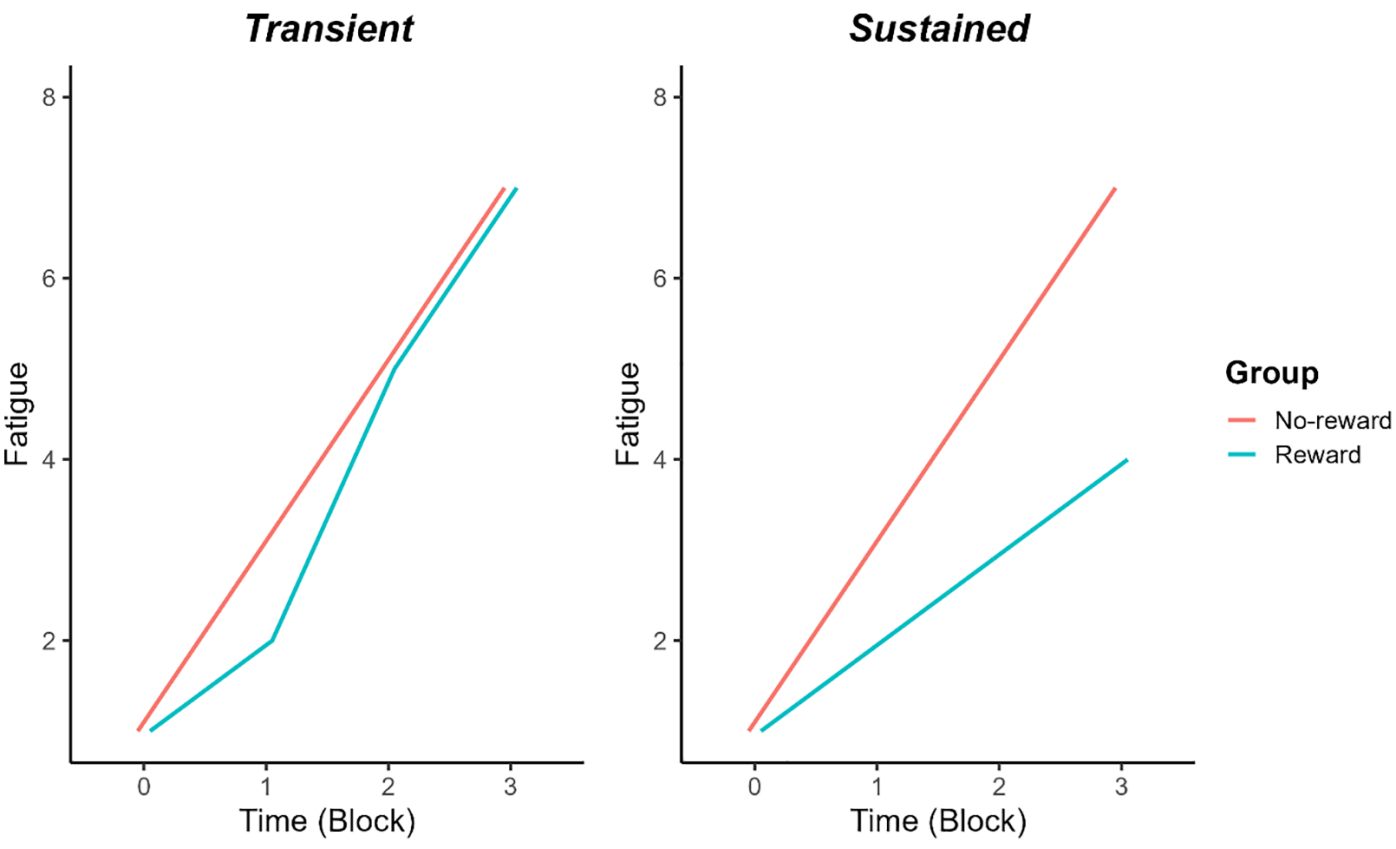
Hypothetical data supporting either a transient (left panel) or sustained (right panel) effect of group (i.e., reward) on perceived fatigue from listening. Block “0” represents baseline fatigue rating. The divergent fatigue scores at Time-point 1 in the “Transient” panel reflect the hypothesised time frame in which fatigue might show a relative (transient) reduction in the “Reward” group before re-converging with the “No-reward” group at Time-point 2.

Current evidence suggests that mental fatigue may be sensitive to motivational factors (Herlambang et al., 2019; Hopstaken et al., 2015). Hopstaken et al. (2015) provided a monetary bonus for accurate working-memory task performance in the final block of their experiment to measure the extent to which a reward incentive could curb the accumulation of mental fatigue. They found that mean fatigue ratings did indeed decrease in the final block, reflecting some recovery from mental fatigue. However, as the monetary incentive was provided in the final experimental block only, the time course of reward effects on perceived fatigue remains unclear. To our knowledge, no studies have monitored the effect of reward on perceived effort and fatigue over the course of a listening task to examine whether reward-based motivation leads to a transient or sustained change in the subjective experiences of effort and fatigue. Figure 1 illustrates two potential hypothetical scenarios in relation to fatigue.

Finally, the studies described above also failed to include an independent measure of current mood state to explore the potential role of emotional processes in modulating perceived effort and fatigue as a function of reward-based motivation. As well as the aforementioned link between mental fatigue and mood (van der Linden et al., 2003), the extent to which an individual experiences a task as subjectively pleasurable has been invoked in FUEL as a factor that may also moderate effortful listening and fatigue (Matthen, 2016; Pichora-Fuller et al., 2016). In other words, listening activities perceived as more rewarding might elicit a more positive mood state (e.g., a sense of contentment from an engaging dialogue) which could in turn diminish the onset of fatigue. In the current study, we aimed to examine associations between perceived effort, mood, and fatigue over time during an effortful listening task in the presence (vs absence) of a monetary reward incentive. We administered a dichotic listening task to simulate a listening scenario with significant cognitive demands, but one in which listening performance would depend critically on the allocation of processing resources (Knight et al., 2023). We hypothesised that:

*Hypothesis 1.* Fatigue ratings in the reward group will be lower overall than fatigue ratings in the no-reward group (Hockey, 2013), with no difference in effort ratings between groups (Koelewijn et al., 2018).
*Hypothesis 2.* Fatigue ratings will show a steeper linear increase in the no-reward group than the reward group, reflecting a sustained (rather than transient) inhibition of fatigue over time owing to continuous reward-based motivation (see Figure 1).
*Hypothesis 3.* Effort ratings will show either a transient effect of reward (i.e., reduced effort after Block 1 only) or no effect of reward on change over time (Koelewijn et al., 2018).
*Hypothesis 4.* The effect of reward on perceived fatigue will be mediated by mood ratings; mood ratings will be overall more positive in the reward than the no-reward group, which will be associated with lower fatigue ratings (Matthen, 2016; van der Linden et al., 2003).

## Method

Hypotheses, methodological plans, and analytic plans for this study were pre-registered (https://osf.io/cvehd/registrations). Experiment stimuli, analysis scripts, raw data, and summary data can be found on our Open Science Framework (OSF) project homepage (https://osf.io/cvehd/). The experiment procedure and materials can also be previewed on Gorilla Open Materials (https://app.gorilla.sc/openmaterials/653834).

### Participants

**Figure 2.**
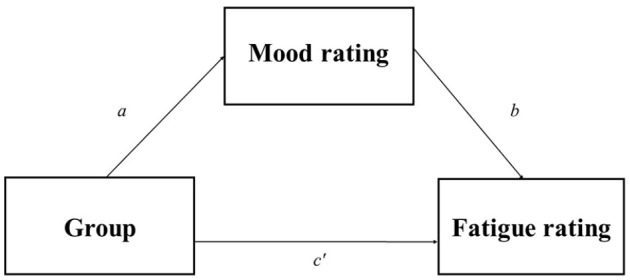
Schematic representation of the variables entered into the mediation analysis. Group (no-reward, reward) was entered as the categorical predictor variable, mood rating (BMIS score) as the mediator variable, and fatigue rating (BFI score) as the dependent variable.

We recruited a total of 200 participants (60 male), aged 18–30 years (*M* = 23.39, *SD* = 3.76). Schoemann et al.’s (2017) “mc_power_med” app was used to calculate sample size requirements for a basic mediation analysis of the hypothesised indirect effect of group (i.e., reward) on fatigue via perceived mood. Figure 2 illustrates the conceptual model tested in the analysis. To calculate sample size requirements, we hypothesised a standardised coefficient of .25 (small-medium effect size) for both the effect of group on mood rating (pathway *a*) and the effect of mood rating on fatigue rating (pathway *b*), and a standardised coefficient of .1 (small effect size) for the direct effect of group on fatigue rating (pathway *c*′).^1^ Using a random seed of 270,488, 1,000 power analysis replications, and 20,000 Monte Carlo draws per replication, and confidence interval (CI) level of 95%, we calculated that a total sample size of 162 (81 per group) would provide the desired statistical power of .80 at α = 0.05 to detect the indirect effect of interest (pathway *ab*). To allow for attrition (given the large number of screening criteria), we recruited 200 participants in total (100 per group).

All participants were recruited via the online recruitment platform Prolific (prolific.co) and financially compensated for their time at a standard rate of £6.50 p/h. We applied the following initial eligibility criteria on Prolific, based on self-reports: (1) Based in the United Kingdom and Ireland, (2) age between 18 and 31 years, (3) English as a first language, (4) normal or corrected-to-normal visual acuity, (5) no known language-related disorders, (6) no diagnoses of mild cognitive impairment or dementia, and (7) a minimum Prolific approval rating of at least 95%. A total of 200 participants met the initial screening criteria on Prolific (100 in each condition). After data collection, participants were excluded if they responded “yes” to any of the screening questions administered at the end of the experiment (details in the “General procedure” section). In total, 15 participants were excluded from the analyses due to being flagged on at least one of the screening checks. In the reward group (*n* = 9), two reported currently suffering from a chronic condition that can cause fatigue; six reported currently taking medication that can cause fatigue; and one reported a hearing loss. In the no-reward group (*n* = 6), one reported currently suffering from a chronic condition that can cause fatigue, and all six reported currently taking medication that can cause fatigue.

All remaining participants scored above chance (i.e., >50%) on the dichotic listening task and were therefore retained in the analyses. A total of 185 participants were entered into the analyses: 94 in the no-reward group and 91 in the reward group. Table 1 shows the demographic breakdown of each group. This study was granted ethical approval by the Departmental Research Ethics Committee at the University of York (ID: 733, year: 2020).

**Table 1.** Demographic information for participants included in the analyses.

	Group	
	*No-reward*	*Reward*
*N*	94	91
Age (years; *M, SD*)	23.61 (3.67)	23.08 (3.75)
Sex (male/female)	33/61	23/68
Study completion time (min; *M, SD*)	24.45 (10.62)	24.73 (7.90)

Study completion time reflects the time taken from when participants began the study to when they returned their completion on Prolific.

### General procedure

We used Gorilla Experiment Builder (www.gorilla.sc; Anwyl-Irvine et al., 2020) to design and host all tasks and rating scales in the main experiment. Participants were recruited on Prolific and directed to Gorilla using the experiment link. On Prolific, participants were instructed to only take part in the experiment if they: (1) had access to a set of headphones or earbuds, (2) could complete the study on a laptop or desktop computer, (3) did not suffer from a known hearing loss in either ear, (4) did not suffer from a chronic condition known to cause fatigue (e.g., chronic fatigue syndrome), (5) were not currently taking medication known to cause fatigue, (6) had not consumed abnormal amounts of a highly caffeinated substance (e.g., coffee) in the last 4 hr, and (7) had a normal night’s sleep (e.g., >6 hr) in the previous night. Participants in both groups completed a series of audio checks before starting the main experiment. First, participants were given the opportunity to play one of the audio stimuli used in the dichotic listening task of the main experiment and adjust the volume to an audible and comfortable level. They then performed a validated headphone check that involved identifying the quietest of three sounds. Importantly, this task can only be performed accurately with the use of stereo headphones (see Woods et al., 2017, for more details). To progress to the experiment, participants were required to accurately identify the quietest sound on at least five of the six trials presented. To allow for potential misunderstanding of the instructions, participants who accurately identified fewer than five trials on the first attempt were given a second opportunity to pass the test. Finally, participants completed a brief “autoplay” check to ensure that their browsers would permit the playback of auditory stimuli during the dichotic listening task. Audio checks lasted approximately 5 min in total.

**Figure 3.**
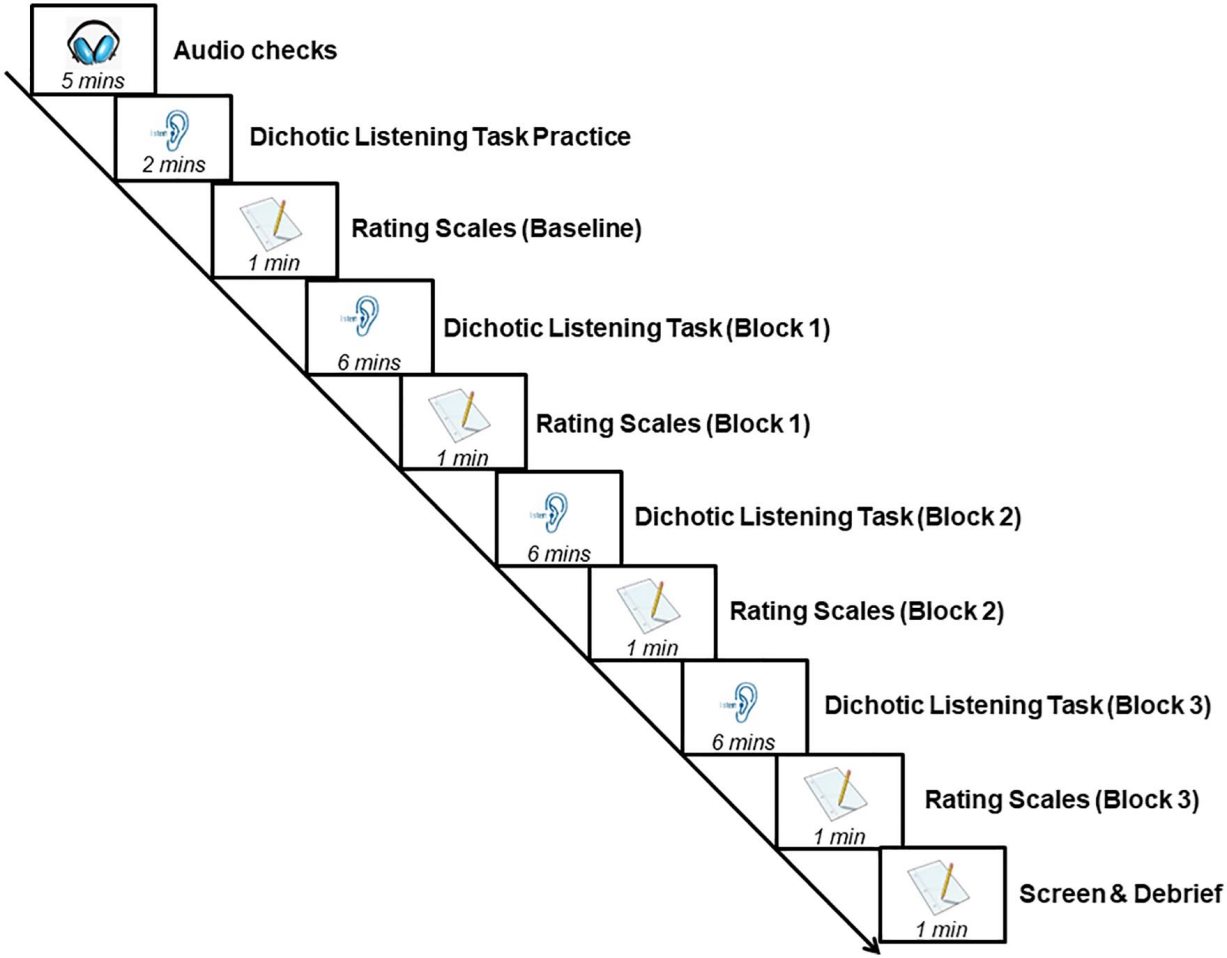
Schematic outline of the study procedure with time estimates for each component. Rating scales included questionnaires measuring perceived effort, mood, and fatigue. Each dichotic listening task block comprised 60 trials.

Following successful completion of the audio checks, participants were given instructions and practised the dichotic listening task. The dichotic listening task practice session consisted of four trials. They then completed each of the three rating scales: perceived effort, mood, and fatigue (details about each scale provided below) in that order. After completing the rating scales, participants performed Block 1 of the dichotic listening task, consisting of 60 trials and lasting approximately 6 min. After completing Block 1, participants once again filled out the three rating scales. This sequence was then repeated for Blocks 2 and 3 of the dichotic listening task. As an additional screening check after completing Block 3 of the dichotic listening task, participants were asked the following five (verbatim) questions, each of which involved a binary (yes/no) response option: (1) Do you currently suffer from a chronic health condition that can cause fatigue (e.g., CFS, cancer, diabetes)? (2) Do you regularly take any medication that can cause fatigue (e.g., antihistamines)? (3) Do you have a known hearing loss in either or both ears and/or regularly use a hearing device (e.g., hearing aid or cochlear implant)? (4) Have you consumed a highly caffeinated substance (e.g., coffee) in the last 4 hr? (5) Did you have a good night’s sleep (e.g., >6 hr) last night? Participants who responded yes to any of Questions 1–3 were removed from the analyses (details in “analyses”). As potential confounds, responses to Questions 4 and 5 were included as covariates in the analyses. Finally, participants were debriefed about the study. The experimental sequence is illustrated in Figure 3.

Participants in both the no-reward and the reward groups completed the same experimental sequence as outlined in Figure 3, with the following exceptions. Participants in the reward group were given the following instructions before performing the dichotic listening task practice:

Before we find out about the listening task, please note that you have an opportunity to gain an additional monetary reward based on your performance accuracy and speed on the listening task. Specifically, for every trial that you perform correctly and in < 2 seconds during the main experiment (i.e., after the practice), you will receive an additional £0.02 on top of your participation payment. As there are 180 trials in total, this means you can earn an additional reward of up to £3.60!

Participants in the no-reward group simply received the message “1^st^/2^nd^/3^rd^ Listening Task complete!” upon completion of each listening block. Participants in the reward group were provided with the following additional information after completing each dichotic listening task block: “Well done! So far, you have earned an additional £**” with the cumulative amount calculated and revealed based on the number of trials responded to correctly in <2 s thus far. Total additional performance-based earnings were given to participants as a bonus payment by the researcher after study completion. The average bonus payment awarded to the participants in the analyses was £3.05 (*SD* = £0.42).

Participants in both conditions took part in the study between the hours of 08:53 am and 12:07 pm within a 3-day testing window. Participants could only take part in the no-reward experiment if they hadn’t already taken part in the reward experiment, and vice versa. In total, the experiment lasted approximately 30 min.

### Stimuli and individual task procedures

#### Dichotic listening task

We used the dichotic listening task developed by Koch et al. (2011) and adapted for use on the Gorilla online platform. For this task, participants heard two digits simultaneously: one in the right ear and one in the left ear. One of the voices was a male voice and the other was a female voice. At the beginning of each trial, a visual text prompt displayed the word “Male” or “Female” (presented centrally on the screen) indicating which voice participants should attend to for that particular trial. The visual prompt remained on screen for 2 s. Immediately after the visual prompt disappeared, the two spoken digits were presented over the headphones. Following presentation of the spoken digits, participants were asked to indicate whether the digit spoken by the attended voice was above or below 5. “Below 5” responses were given by pressing “f” with the left index finger and “above 5” responses were given by pressing “j” with the right index finger. Participants were given visual prompts for these two response options on the left (press “f”) and right (press “j”) side of the screen. Presentation of the visual prompts was synchronised with the onset of the spoken digits. Participants were asked to respond as quickly and accurately as possible, and were given four practice trials to familiarise themselves with the task.

All dichotic spoken digits were edited in Audacity to include matching silent onsets lasting 200 ms. Audio files for digits 1–9 (excluding 5) were created using a free online text-to-speech MP3 creator (www.ttsmp3.com). MP3 files were created in both a male and a female voice. Of the default options on the website, we used the British male voice “Brian” and British female voice “Emma.” Each audio file had a sampling rate of 48 kHz. These files were then combined in Audacity to create stereo dichotic stimuli. Participants performed 180 experimental trials in total; 60 trials in each of three listening blocks. Within each block, an equal number (30) of “female” and “male” prompts were administered. Of the 30 “female” and 30 “male” prompt trials in each block, half (i.e., 15/30) were “congruent” trials, in which both spoken digits were either above or below 5. The other half were “incongruent,” in which one digit was above 5 and the other below 5. The same digits were never presented together in a given trial. The number of “above 5” and “below 5” correct response trials was balanced (i.e., 30 each) within each block. The lateral position of the female and male voice was also counterbalanced within each block (i.e., the female voice was presented to the left ear on 30 trials, and vice versa). The order of stimuli presentation was fully randomised within each block.

#### Perceived effort rating

Perceived effort ratings were collected based on an adapted version of the NASA task load index item assessing mental demand (Hart & Staveland, 1988), a commonly used subjective measure of effort (Dimitrijevic et al., 2019; McGarrigle & Mattys, 2023; Strand et al., 2018). Specifically, we asked “How hard did you have to work to accomplish your level of performance (speed AND accuracy) in the listening task? (EFFORT)” (100-step scale from *very low effort* to *very high effort*). Participants provided responses using an on-screen slider bar with values ranging from 0 to 100 in increments of 1. A circular icon was positioned on the midpoint of the scale (50) to begin with and participants adjusted the icon using a mouse, with verbal anchors positioned at each endpoint of the slider scale. A “Next” box was positioned at the bottom of the screen which participants clicked on to advance to the next stage of the experiment.

#### Perceived mood rating

The Brief Mood Introspection Scale (BMIS) was used to collect perceived mood ratings (Mayer & Gaschke, 1988). In the BMIS, participants are provided with a list of 16 adjectives (e.g., “lively,” “sad,” “gloomy”) and asked to circle one of four categorical response options ranging from *definitely do not feel* (coded as “1”) to *definitely feel* (coded as “4”) to indicate how well each adjective describes their present mood. A “Next” box was positioned at the bottom of the screen which participants clicked on to advance to the next stage of the experiment.

#### Perceived fatigue rating

Perceived fatigue ratings were collected using an item from the Brief Fatigue Inventory (BFI) scale (Mendoza et al., 1999), an instrument used to quickly assess fatigue severity. Specifically, participants were asked to “Please rate your fatigue (weariness, tiredness) by selecting the one number that best describes your fatigue right NOW.” This question was chosen because it assessed fatigue “right now,” whereas the other items on the scale assessed fatigue over a 24-hr period and would therefore not be suitable for measuring acute changes over time during a listening task. Participants provided responses using an on-screen slider bar with values ranging from 0 to 10 in increments of 1. A circular icon was positioned on the midpoint of the scale (5) to begin with and participants adjusted the icon using a mouse, with verbal anchors (*no fatigue* to *as bad as you can imagine*) positioned at each endpoint of the slider scale.

### Analysis

#### Dichotic listening task data pre-processing

Individual trial response times (RTs) in the dichotic listening task that exceeded 3 *SDs* below or above the mean RT for each participant were removed from the dataset. This resulted in the removal of 284 trials in the no-reward group (1.7% of responses) and 262 trials in the reward group (1.6% of responses). The highest number of trials removed for a single participant was 7/180 (3.9%). To limit the influence of trials for which there may have been lapses in concentration or misperceptions, RTs were analysed for correct responses only. Given the generally high level of performance across both groups (>90%), only 7% of the remaining trials were removed from the RT analysis due to incorrect responses.

#### Rating scales

Scores on the NASA perceived effort scale ranged from 0 to 100, with higher scores reflecting increased perceived effort. Total scores on the BMIS perceived mood scale ranged from 16 to 64, with higher scores reflecting more pleasant perceived mood ratings. Of the 16 items on the BMIS scale, 8 were negative/unpleasant items (e.g., “gloomy,” “grouchy”) and were therefore recoded to ensure that higher total scores reflected more pleasant mood ratings. Scores on the BFI perceived fatigue scale ranged from 0 to 10, with higher scores reflecting increased perceived fatigue. For all three rating scales, mean scores were calculated as a function of group (no-reward, reward) and block (0, 1, 2, 3) with block level “0” reflecting the baseline rating collected immediately after the practice trials.

#### Mixed-effects models

We used the “lme4” package (Bates et al., 2015) in R Studio (R version 4.2.3; R Development Core Team, 2023) to examine the effects of group (no-reward, reward) and block (0, 1, 2, 3) on each outcome variable: (1) dichotic listening performance accuracy, (2) dichotic listening RT, (3) effort rating, (4) mood rating, and (5) fatigue rating. Plots were created using the “ggplot2” package (Wickham, 2016). Performance accuracy on the dichotic listening task was coded as a binary outcome variable (1 = *correct*, 0 = *incorrect*). A Generalised Linear Mixed-effects Model (GLMM) was therefore used for analysis of the accuracy data. A binomial response distribution was specified in the GLMM with a “logit” link function. RTs and responses to each of the three rating scales (effort, mood, and fatigue) were analysed using four separate Linear Mixed-effects models (LMMs). For all the above analyses, the between-subjects categorical variable “group” (reward, no-reward) was modelled as a fixed effect. Binary responses (0 = *no*, 1 = *yes*) to the “caffeine” screening question (“Have you consumed a highly caffeinated substance (e.g., coffee) in the last four hours?”) and “sleep” screening question (“Did you have a good night’s sleep (e.g., >6 hours) last night?)” were included as covariates in each model.

The within-subjects continuous variable “block” was also included in each model as a fixed effect. Although the models for dichotic listening data (accuracy and RT) included block with three levels (1, 2, 3), the models for analysis of the rating scales data (effort, mood, and fatigue) included an additional level to account for the baseline rating score. Thus, in the rating models, block was coded with four levels (1, 2, 3, 4) with “1” representing the baseline score. By-subject intercepts and block slopes were included as random effects in each model to account for inter-individual variance in both the overall score (intercept) and change over time (block slope) for each outcome variable. To account for by-item variance in the dichotic listening (accuracy, RT) models, we included an intercept term for the individual items (i.e., auditory stimuli).^2^

Likelihood ratio tests were conducted to determine whether the fixed effects and interactions contributed significantly to the model. To conduct these tests, we used the “mixed” function from the “afex” package (Singmann et al., 2023), which converts variables in the model from default dummy coding (0, 1) to sum-coding (–1, 1). Fixed effects in the model can therefore be interpreted as main effects (i.e., the effect of one variable holding other variables constant), rather than simple effects (i.e., the effect of one variable but only on a specific level of another variable). R syntax for each final model can be found on our OSF project page (https://osf.io/cvehd/).

#### Mediation analysis

Mediation analysis was conducted to test our hypothesis regarding the indirect effect of group on fatigue via mood. This analysis was conducted using the PROCESS (Hayes, 2017) macro on SPSS v25. We entered group as the categorical predictor variable, mood rating as the mediator variable, and fatigue rating as the outcome variable. Figure 2 illustrates the conceptual model tested in the analysis. As with the mixed-effects model analyses, binary responses to the “caffeine” and “sleep” screening questions were included as covariates. Baseline mood and fatigue ratings were also entered into the model as covariates to control for the effect of baseline differences in mood and fatigue ratings. CIs were derived from 5,000 bootstrap samples using a random seed generator of 270,488. Following the recommendations of Hayes (2017), direct and indirect effects were deemed statistically significant if both bootstrap CIs were either entirely above or below zero.

## Results

### Dichotic listening task performance accuracy and RT

**Figure 4.**
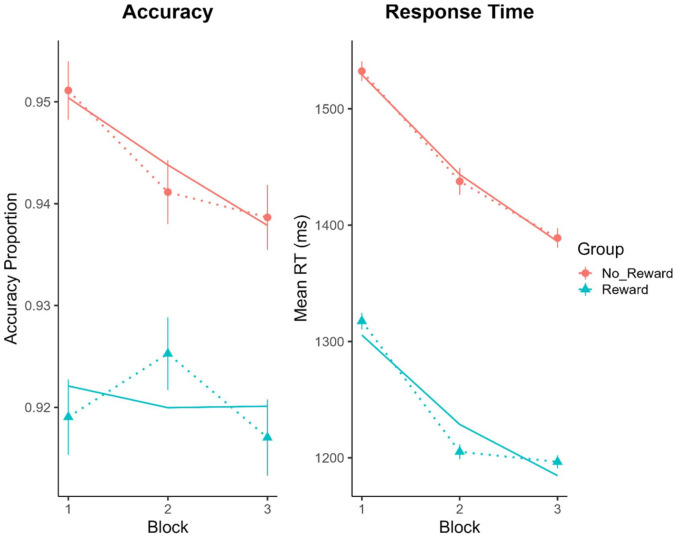
Mean proportion correct (left panel) and RT (right panel) with ±*SE* bars on the dichotic listening task as a function of block (1–3) and group (no-reward, reward). Overlaid solid lines illustrate the GLMM (accuracy) and LMM (RT) model fits to the data.

Figure 4 displays the mean dichotic listening task performance accuracy and RT as a function of group and block. GLMM analyses revealed that there was a significant effect of group on accuracy (χ^2^ (1, *N* = 185) = 8.04, *p* = .005), with better performance in the no-reward than the reward group. There was no effect of block (χ^2^ (1, *N* = 185) = 0.87, *p* = .35) nor any interaction between group and block (χ^2^ (1, *N* = 185) = 1.07, *p* = .30) on accuracy.

LMM analyses revealed a significant main effect of group on RTs (χ^2^ (1, *N* = 185) = 19.24, *p* < .001), with slower RTs in the no-reward than reward group. There was also a significant effect of block (χ^2^ (1, *N* = 185) = 45.00, *p* < .001) with RTs becoming faster as the experiment progressed. There was no significant interaction between group and block (χ^2^ (1, *N* = 185) = 0.71, *p* = .40).

### Perceived effort, mood, and fatigue ratings

**Figure 5.**
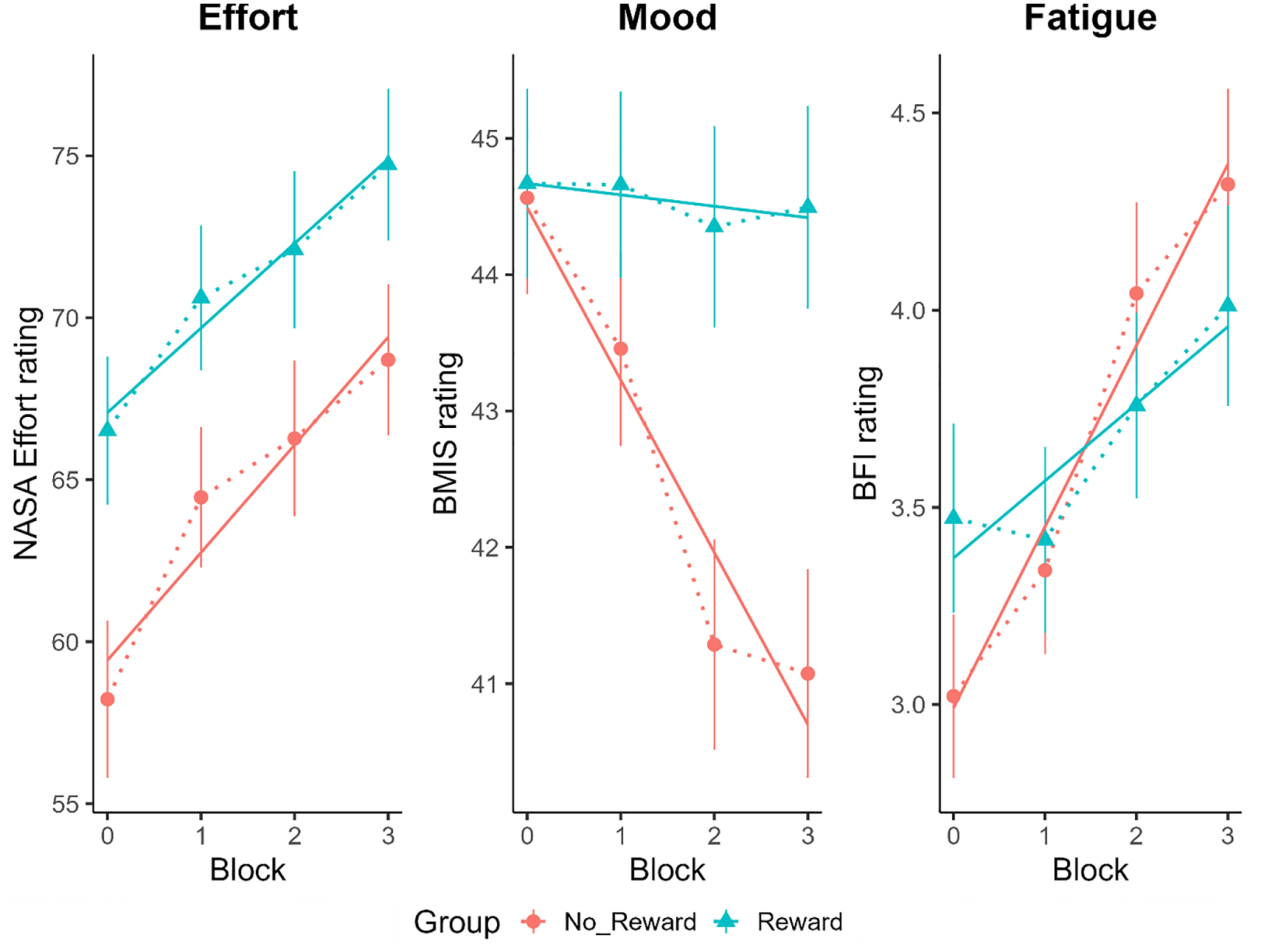
Mean ratings for perceived effort (left panel), mood (middle panel), and fatigue (right panel) with ±*SE* bars as a function of block and group. Block “0” represents the mean baseline rating score provided immediately after the practice trials. Overlaid solid lines illustrate the LMM model fits to the data. NASA effort ratings range from 0 to 100, with higher scores reflecting increased perceived effort. BMIS ratings range from 16 to 64, with higher scores reflecting a more pleasant perceived mood state. Finally, BFI ratings range from 0 to 10, with higher scores reflecting increased perceived fatigue.MIS:: Brief Mood Introspection Scale.FI:: Brief Fatigue Inventory; LMM: Linear Mixed-effects Model.

Figure 5 displays the mean perceived effort, mood, and fatigue ratings as a function of group and block. We found a significant effect of group on perceived effort (χ^2^ (1, *N* = 185) = 5.35, *p* = .02), with higher perceived effort in the reward compared with the no-reward group. There was also a significant effect of Block on perceived effort (χ^2^ (1, *N* = 185) = 35.59, *p* < .001) with effort ratings generally increasing as a function of time-on-task. There was no significant interaction between group and block (χ^2^ (1, *N* = 185) = 0.57, *p* = .45).

We found no significant effect of group on mood ratings (χ^2^ (1, *N* = 185) = 1.42, *p* = .23). There was, however, a significant main effect of block (χ^2^ (1, *N* = 185) = 27.11, *p* < .001) and a significant interaction between group and block (χ^2^ (1, *N* = 185) = 21.15, *p* < .001). Although mood ratings generally plateaued for participants in the reward group, there was a more pronounced linear decrease in mood ratings as a function of time-on-task for participants in the no-reward group.

We found significant effects of group and block on fatigue ratings (χ^2^ (1, *N* = 185) = 4.56, *p* = .03; χ^2^ (1, *N* = 185) = 44.32, *p* < .001, respectively). There was also a significant interaction between group and block (χ^2^ (1, *N* = 185) = 7.96, *p* = .005). Although participants in both the reward and no-reward groups showed a general increase in fatigue as a function of time-on-task, this increase was relatively steeper in the no-reward versus the reward group.

### Mediation analysis

Table 2 shows the correlations between all five variables when scores are collapsed across the three experimental blocks. We conducted a mediation analysis to examine the hypothesis that perceived mood would mediate the effect of group on perceived fatigue ratings (cf. Figure 2). We found an indirect effect of group on perceived fatigue via perceived mood. Specifically, participants in the no-reward group were significantly more likely to report lower (i.e., more unpleasant) mood ratings overall (*a* = –2.49, *p* < .001), and individuals who provided lower mood ratings were more likely to also provide higher perceived fatigue ratings (*b* = –0.12, *p* < .001). Bootstrap CIs for the indirect effect (*ab* = 0.30) were entirely above zero (0.16–0.47). There was no significant direct effect of group on perceived fatigue rating as the bootstrap CIs straddled zero (*c*′ = 0.19, bootstrap CIs = [–0.14, 0.53]).

**Table 2.** Correlation coefficients between all variables.

	Effort	Mood	Fatigue	DL_Accuracy	DL_RT
Effort	—				
Mood	–.17*	—			
Fatigue	.16*	–.52**	—		
DL_Accuracy	.13	–.008	–.005	—	
DL_RT	.09	–.15*	.02	–.18*	—

DL_Accuracy: percentage correct on dichotic listening task; DL_RT: mean correct response time on dichotic listening task.

* *p* < .05. **p < .01.

### Exploratory mediation analysis

We conducted an additional mediation analysis to examine the alternative hypothesis that reward affected perceived fatigue which in turn altered mood ratings. Fatigue ratings were this time entered as the “mediator” variable and mood ratings as the “outcome” variable. All other aspects of the analysis were identical to the original mediation model. This analysis revealed an indirect effect of reward group on mood ratings via perceived fatigue (*ab* = –0.59, bootstrap CIs = [–1.00, –0.21]). Participants in the no-reward group were significantly more likely to report higher fatigue ratings overall (*a* = 0.50, *p* = .005), and individuals who provided higher fatigue ratings were more likely to provide lower (more unpleasant) mood ratings (*b* = –1.18, *p* < .001). However, there was also a significant direct effect of group on mood rating (*c*′ = –1.90, bootstrap CIs = [–2.92, –0.89]).

## Discussion

The present study examined the effect of reward-based motivation on changes over time in perceived effort, mood, and fatigue. First, we hypothesised that fatigue ratings would be lower in the reward than the no-reward group reflecting reward-based inhibition of mental fatigue, but that there would be no overall differences between groups in perceived effort (Hypothesis 1). Hypothesis 1 was partially supported; overall perceived fatigue ratings were lower in the group who received a monetary incentive, but perceived effort was also higher in this group than in the no-reward group. Second, we predicted that fatigue ratings would show a sustained linear increase over time which would be more pronounced in the no-reward group (Hypothesis 2). We found support for this hypothesis, with results showing greater accumulation of mental fatigue in the unrewarded listening condition. On the contrary, we hypothesised that changes over time in effort would show either a transient effect of reward or no effect at all (Hypothesis 3). And indeed, although effort ratings did show an increase over time, this change did not interact with the absence/presence of monetary reward, supporting Hypothesis 3. Finally, we predicted that mood ratings would mediate the effect of reward on perceived fatigue (Hypothesis 4). Mediation analysis supported this hypothesis, demonstrating: (1) evidence for an indirect effect of reward on perceived fatigue via mood ratings and (2) no evidence for a direct effect of reward on perceived fatigue when mood ratings were statistically controlled.

The current study provides novel evidence for a differential impact of reward-based motivation on perceived effort versus fatigue. Specifically, results highlight a scenario in which listening is perceived to be more effortful yet shielded from the onset of mental fatigue over time. The effect of reward on perceived fatigue became more pronounced as the task progressed, suggesting a gradual but more pronounced accumulation of fatigue during unrewarding listening challenges. Feedback at the end of each block on how much monetary reward had been accumulated may have contributed to this sustained inhibition of perceived fatigue in the reward group. Previous research suggests that performance feedback may help to increase task engagement and motivation (Salmoni et al., 1984) and thus help to reduce mental fatigue (Herlambang et al., 2019). Higher overall perceived effort ratings in the “reward” group support previous literature showing that young adults are generally more willing to engage cognitive resources during listening if doing so can result in a monetary gain (McLaughlin et al., 2021). The differential effects of reward-based motivation on perceived effort and fatigue are consistent with both FUEL (Pichora-Fuller et al., 2016) and MCT (Hockey, 2013) by illustrating that the experience of effort may not result in mental fatigue if the effort investment is deemed sufficiently valuable. Nonetheless, although both theoretical accounts highlight the role of motivation during effortful listening (FUEL) and mental fatigue (MCT), subjective perceptions of effort and fatigue are often described synonymously. The current study shows that perceived effort and fatigue are underpinned by different mechanisms.

Links between an individual’s current mood state and their propensity to experience mental fatigue have been demonstrated in previous research (Leavitt & DeLuca, 2010; McGarrigle, Knight, et al., 2021; van der Linden et al., 2003). However, the extent to which mood state may govern the effect of reward-based motivation on perceived fatigue from listening has not yet been the focus of systematic examination. The current study revealed an indirect effect of reward on perceived fatigue via mood ratings; individuals who completed the listening task with a monetary incentive indicated more pleasant mood ratings overall which, in turn, was associated with reductions in the experience of fatigue. Importantly, there was no direct effect of reward on perceived fatigue independent of mood ratings. This suggests that a mechanism by which reward-based motivation inhibits the onset of listening-related fatigue is by improving one’s mood state during task completion. Interestingly, although baseline mood ratings were similar in both the no-reward and the reward groups, perceived mood showed a clear progressive decline over time in the no-reward group, whereas monetary reward resulted in more stable (and pleasant) mood ratings over time in the reward group. These findings support the MCT (Hockey, 2013) characterisation of mental fatigue as a fundamentally emotional response that instigates a cost–benefit analysis of goal pursuit. These findings also support Matthen’s (2016) assertion that outcomes relating to effortful listening may vary according to how much pleasure or value is derived from the process of listening.

Although the listening task performance and RTs were not primary outcomes of interest in the current study, some discussion of these findings is warranted. Despite being instructed to prioritise both accuracy and speed (i.e., they could only earn bonus money for trials performed correctly and in less than 2 s), the monetary incentive seems to have induced a speed–accuracy trade-off in the reward group; performance accuracy was significantly worse in this group but responses were significantly faster. One possibility is that, because performance accuracy was generally very high (>90%) in both groups, participants in the reward group felt that prioritising response speed over accuracy would be a more productive response strategy. Indeed, the literature suggests that individuals will often trade off in this manner if it serves to maximise reward benefit (Bogacz et al., 2010).

As mediation analysis is a correlational approach, determining the precise sequence of effects in the path model is not straightforward. In other words, although our analysis supports the interpretation that reward affected mood ratings, which in turn affected perceived fatigue, another interpretation is possible; that reward affected perceived fatigue which in turn altered mood ratings. To statistically test for this alternative hypothesis, we conducted an additional exploratory mediation analysis, this time with fatigue ratings entered as the “mediator” variable and mood ratings as the “outcome” variable. This analysis revealed an indirect effect of reward group on mood ratings via perceived fatigue. However, importantly, this time there was also a significant direct effect of group on mood rating. Therefore, participants in the reward group were significantly more likely to provide more pleasant mood ratings, irrespective of perceived fatigue. The strong evidence for a direct effect of reward on mood ratings, and the lack of a direct effect of reward on perceived fatigue independently of mood ratings, supports the hypothesised model in Figure 2 as the most plausible path sequence.

Mean fatigue scores did not exceed 5 (out of 10) in either group, even at the end of the final block of trials, suggesting that most participants did not reach their mental fatigue threshold by the end of the experiment. However, it is clear that mental fatigue was elicited to an extent that was sufficient to reveal both differences as a function of monetary reward and meaningful changes over time. Examining the relationship between perceived effort, mood, and fatigue in situations where mental fatigue is more exacerbated may provide insight into the mechanisms that underlie more severe cases of fatigue (e.g., in individuals with a chronic illness). To simulate a challenging and effortful listening experience, we used a dichotic listening task in the current study. However, one limitation of this approach is that it involves responding to a closed-set sequence of digits only, thus limiting the extent to which the stimuli can resemble everyday listening experiences which typically involve more complex language operations. Use of more naturalistic stimuli in future research may help to shed light on the cognitive processes that underlie more routine experiences of effortful listening. Furthermore, rather than using monetary reward to increase motivation, varying the intrinsic value of cognitive engagement (e.g., by tailoring speech materials to match the interests of individual participants) might help to reveal the dynamic interplay between effort, mood, and fatigue during listening.

## Conclusion

The current findings shed light on the complex relationships between motivation, effort, mood, and mental fatigue during listening. We report evidence for differential effects of reward-based motivation on perceived effort and fatigue ratings which highlight their distinct nature. We also provide novel evidence that changes to one’s mood state represent a mechanism by which perceived fatigue may be inhibited (or elicited) during effortful listening which may be used to inform interventions for individuals who suffer from listening-related fatigue.

## Notes

1. Note that the apostrophe (*c*′) denotes the fact that this path represents the effect of *X* (Group) on *Y* (Fatigue) although controlling for M (Mood), as opposed to the total effect which is commonly represented without an apostrophe and includes the indirect effect.

2. As rating scale responses were not made to specific items/stimuli, by-item random effects were not included in the rating scale LMMs.
